# Functional Stability and Community Dynamics during Spring and Autumn Seasons Over 3 Years in Camargue Microbial Mats

**DOI:** 10.3389/fmicb.2017.02619

**Published:** 2017-12-22

**Authors:** Mercedes Berlanga, Montserrat Palau, Ricardo Guerrero

**Affiliations:** ^1^Department of Biology, Environment and Health, Section Microbiology, Faculty of Pharmacy and Food Sciences, University of Barcelona, Barcelona, Spain; ^2^Laboratory of Molecular Microbiology and Antimicrobials, Department of Pathology and Experimental Therapeutics, Faculty of Medicine, University of Barcelona – Institut d’Investigació Biomédica de Bellvitge, Barcelona, Spain; ^3^Academia Europaea-Barcelona Knowledge Hub, Barcelona, Spain

**Keywords:** Camargue microbial mats, 16S rRNA amplicon sequencing, shotgun metagenome, diversity, functionality

## Abstract

Microbial mats are complex biofilms in which the major element cycles are represented at a millimeter scale. In this study, community variability within microbial mats from the Camargue wetlands (Rhone Delta, southern France) were analyzed over 3 years during two different seasons (spring and autumn) and at different layers of the mat (0–2, 2–4, and 4–6 mm). To assess bacterial diversity in the mats, amplicons of the V1–V2 region of the 16S rRNA gene were sequenced. The community’s functionality was characterized using two approaches: (i) inferred functionality through 16S rRNA amplicons genes according to PICRUSt, and (ii) a shotgun metagenomic analysis. Based on the reads distinguished, microbial communities were dominated by Bacteria (∼94%), followed by Archaea (∼4%) and Eukarya (∼1%). The major phyla of Bacteria were Proteobacteria, Bacteroidetes, Spirochaetes, Actinobacteria, Firmicutes, and Cyanobacteria, which together represented 70–80% of the total population detected. The phylum Euryarchaeota represented ∼80% of the Archaea identified. These results showed that the total bacterial diversity from the Camargue microbial mats was not significantly affected by seasonal changes at the studied location; however, there were differences among layers, especially between the 0–2 mm layer and the other two layers. PICRUSt and shotgun metagenomic analyses revealed similar general biological processes in all samples analyzed, by season and depth, indicating that different layers were functionally stable, although some taxa changed during the spring and autumn seasons over the 3 years. Several gene families and pathways were tracked with the oxic-anoxic gradient of the layers. Genes directly involved in photosynthesis (KO, KEGG Orthology) were significantly more abundant in the top layer (0–2 mm) than in the lower layers (2–4 and 4–6 mm). In the anoxic layers, the presence of ferredoxins likely reflected the variation of redox reactions required for anaerobic respiration. Sulfatase genes had the highest relative abundance below 2 mm. Finally, chemotaxis signature genes peaked sharply at the oxic/photic and transitional oxic-anoxic boundary. This functional differentiation reflected the taxonomic diversity of the different layers of the mat.

## Introduction

Extant microbial mats are valid equivalents of some of the Earth earliest Archaean ecosystems, which form lithified (e.g., Shark Bay, Western Australia) as well as non-lithified (e.g., Ebro Delta, north-eastern Spain) structures (Dupraz and Visscher, 2005; Wierzchos et al., 2006; Ruvindy et al., 2016). Among their features is their visible lamination, result of physicochemical gradients (e.g., light, oxygen and sulfide) along the vertical axis that allows the creation of microenvironments at a millimeter scale within the mat and justify its taxonomically and functionally heterogeneity (Guerrero and Berlanga, 2013; Harris et al., 2013; Wong et al., 2015; Saghaï et al., 2017). Microbial mats contain diverse groups of microorganisms, such as producers (e.g., photosynthetic bacteria), heterotrophs (e.g., aerobic/anaerobic respirators, especially sulfate-reducing species, and fermenters), and chemolithotrophs (notably, sulfur-oxidizing species) (Bolhuis et al., 2014).

Microorganisms do not exist in isolation (as axenic culture) but form complex ecological interaction webs, such as food-webs, by combining metabolic pathways flows (Faust and Raes, 2012; Sachs and Hollowell, 2012; Guerrero and Berlanga, 2016). Microbial mats are an extraordinary example of microbial interaction, where all possible connections (commensalism, mutualism, competition, predation, or parasitism) among microorganisms may be possible. Elucidating competitive and cooperative relationships is a challenge in describing a microbial interaction network, and interpretation of such networks is not straightforward. Population interactions, such as metabolic, physical, or signaling regulations, may determine temporal changes in the composition, function, or spatial organization of the microbial community (Widder et al., 2016). Modeled networking is a versatile tool for predicting relationships that can be due to genes (Christian et al., 2007; Großkopf and Soyer, 2014) or OTUs’ presence/absence and abundance (Weiss et al., 2016). Those models can generate hypotheses on what interactions could be biologically relevant. In addition, interactions may be studied through laboratory experimental work. For instance, Long et al. (2012) tested antagonistic interaction between heterotrophic bacteria isolates from microbial mats as regulators of the community structure.

According to Liebig’s law of the minimum, growth is regulated by the amount of the scarcest nutritional element available; thus, among biotic conditions, the availability of food regulates microorganismal biomass (Guerrero and Berlanga, 2006). According to Shelford’s law of tolerance, each organism requires certain abiotic conditions to survive and develop (Guerrero and Berlanga, 2006). The abiotic factors influencing the distribution and function of microbial populations are principally the diel fluctuations in the concentrations of oxygen, sulfide, and other chemical nutrients and the cyclic seasonal fluctuations of inundating and desiccation (Bolhuis et al., 2014). During the day, in microbial mats can be distinguished three main chemical zones: the oxic/photic (∼0–2 mm depth) zone, the low-sulfide or transitional oxic-anoxic zone (∼2–4 mm depth), and the high-sulfide/anoxic zone (∼5 mm and deeper). At night, however, the mats become anoxic and high in hydrogen sulfide concentration, as a result of continuing sulfate reduction in the absence of oxygenic photosynthesis (Ley et al., 2006; Villanueva et al., 2007; Nielsen et al., 2015; Guerrero and Berlanga, 2016).

Microbial mats are present in several habitats such as coastal zones (e.g., Guerrero Negro, Baja California, Mexico), athalassic wetlands (e.g., Salar de Atacama, north of Chile), diverse geothermal environments (hot springs), and in polar regions. Camargue and Ebro Delta microbial mats are coastal estuarine not lithified mats from the Western Mediterranean. Camargue microbial mats were usually permanently flooded and contained more salinity concentration than Ebro Delta mats, although season temperature and latitude were similar in both microbial mats (Berlanga et al., 2008). The microbial mats in the area of Salins-de-Giraud, in the Camargue (04° 11′ E to 04° 57′ E; 43° 40′ N to 44° 40′ N), are located inside commercial salterns, which are being mined for salt. These salterns are a succession of water concentration ponds at the final part of the main mouth of the Rhone River. In the first series of ponds, seawater is concentrated to a total salinity of 50–130‰. This pond has a depth of the water column that never exceed 20 cm. In the second series, water is concentrated to salinities in the range of 130–300‰, while in the final series of ponds the salinity is increased to 340–350‰ (Fourçans et al., 2004; Guerrero and Berlanga, 2013). The vertical structure and temporal variation of microbial mats from the Camargue were previously revealed by combining molecular approaches, lipid analyses, and microscopy (Fourçans et al., 2004, 2008; Villanueva et al., 2007; Berlanga et al., 2008).

The aim of this study was to decipher the phylogenetic composition of the Camargue microbial mat community and to interpret its functional potential complexity using next-generation sequencing (NGS) methods at temporal level through three consecutive years (two season) at the same sampling place. The NGS studies used in this work included amplicon sequencing (for variant identification and phylogenetic surveys) and random-genome shotgun sequencing (for metagenomics analysis). Core samples of microbial mats from the Camargue were analyzed in detail over 3 years (2011–2013), during two different seasons, spring and autumn, and at different layers (0–2 mm, 2–4 mm, and 4–6 mm) to study the community variability of the mat.

Winter and summer seasons in the area of Salins-de-Giraud, in the Camargue had “extreme” temperature conditions, colder and warmer respectively, when compared to spring and autumn seasons. We supposed that spring and autumn had “transitional” conditions respect to temperature between those extremes. Indeed, temperature between spring and autumn in analyzed years was similar. Salinity in Camargue microbial mats was similar in all seasons through years analyzed (55–65‰). During winter, ambient temperatures are lower and daily temperature variations (day–night) are less pronounced than in summer, so less pronounced daily temperature variation in winter may have favored the adaptation of the microbial population to lower temperatures. We speculated that if there were changes in the microbial composition by temperature (cold or warm), it would be interesting to study if microbial communities could reach similar populations in spring and autumn seasons, although the initial population may be different from populations “cold adapted” in winter and populations “warm adapted” in summer. Results could reflect the “capacity of resilience” of the Camargue microbial mat system after a perturbation such as a cold period (observed on spring samples) and after a warm period (autumn samples). The adaptation of populations to different temperatures may help to provide homeostasis within a mat community (Ward et al., 2006; Wieland and Kühl, 2006; Berlanga et al., 2008). In addition, the results will shed light on how shifts in community taxonomy may affect the relationship between biodiversity and ecosystem function. As such, they significantly enhance our understanding of the community structure of the Camargue microbial mats, their contributions to element cycling and other fundamental processes that are ongoing within the mat that are critical to the function of this ecosystem.

## Materials and Methods

### Sample Collection

Samples analyzed in this study were collected at noon (12.00 h) in May (spring, SP) and November (autumn, AU) during three consecutive years (2011–2013). Environmental temperatures in May and November ranged from 15–18°C and 13.5–15°C, respectively. The mats in all cases were flooded. The salinity of the water covering the mats was 58–62‰ in May and 55–65‰ in November. Mat samples were collected in cores (1 cm × 3 cm) and frozen in liquid nitrogen immediately. Then, cores were stored in the lab at -80°C until DNA extraction. We collected three cores as in previous works (Armitage et al., 2012; Harris et al., 2013), separated by 10 cm each, for each year and season. Our samples were taken each year at the same location. Dillon et al. (2009) sampled cores across 1 km. They observed that population structure diverged with increasing distance between sample sites, but positional replicates were highly similar among samples < 1 m distance. We pooled the extracted DNA for the three samples corresponding to year/season and layer to obtain a representative sample for each year/season/layer. We expected that if there were differences in microbial composition it could be due to seasonal environmental variables and not to location of sampling.

### DNA Extraction and Amplification

The frozen cores were sliced with a sterile blade in aseptic conditions horizontally in 2-mm increments (from the top to a depth of 6 mm): 0–2 mm (layer 1, oxic/photic layer), 2–4 mm (layer 2, oxic-anoxic transition layer), and 4–6 mm (layer 3, anoxic layer). A fresh blade was used at each interface. Then, pieces of microbial mat of approximately 3 mm^3^ were cut from each slice and suspended in 100 μl of TE buffer in 2.0-ml vials containing a capful of 0.1-mm glass beads. The mixture was homogenized for 1 min in a Mini-BeadBeater-8 (Biospec Products, Bartlesville, OK, United States), and centrifuged at high speed for 2 min. While avoiding transfer of the beads, ∼500 μl from each sample was pipetted into sterile 1.5-ml Eppendorf tubes. DNA was extracted using a phenol-chloroform mixture and precipitated in the cold using 95% ethanol. Three DNA extractions corresponding to every year, season and layer were performed. The DNAs obtained were mixed to correct for potential local heterogeneity effects to obtain a representative sample for each year, season and layer.

For years 1, 2, and 3, we performed amplicon sequencing of the bacterial 16S rDNA gene. The primers used for multiplex Roche 454 GS FLX pyrosequencing, contained a 25 nucleotide sequence adapter, 10-base-pair molecular barcode (multiplex identifier), and the universal bacterial sequence for the region V1–V2, 8F-338R (5′-AGAGTTTGATCCTGGCTCAG-3′ and 5′-TGCTGCCTCCCGTAGGAGT-3′) (Armitage et al., 2012; Harris et al., 2013; Yang et al., 2013). We used three different barcodes (each one for 0–1 mm, 2–4 mm, and 4–6 mm; ACGAGTGCGT, ACGCTCGACA, AGACGCACTC, respectively). Samples analyzed were: SP1-1, SP1-2, SP1-3; AU1-1, AU1-2, AU1-3; SP2-1, SP2-2, SP2-3; AU2-1, AU2-2, AU2-3; SP3-1, SP3-2, SP3-3; AU3-1, AU3-2, AU3-3 (SP and AU indicated the season analyzed; the first number, the year, and the second number, the layer). A PCR from each DNA was performed. The cycling conditions were 94°C for 3 min, followed by 30 cycles of 94°C for 30 s, 56°C for 40 s, 68°C for 40 s, and a final extension step at 68°C for 6 min. The resulting product was checked for size and purity on an agarose-SYBR Safe DNA gel that was subsequently stained (Invitrogen, San Diego, CA, United States). The amplicons were purified using a Pure Link kit (Invitrogen, San Diego, CA, United States) and quantified using Qubit and Bioanalyzer (Berlanga et al., 2016). A pool of amplicons was mixed in equimolar amounts (e.g., spring 1st year, amplicons obtained for 0–2, 2–4, and 4–6 mm), and then prepared for 454-pyrosequencing according to the manufacturer’s instructions. Pyrosequencing coverage (depth sequencing) resulted in 99,216 total raw reads that after quality control processing resulted in 44,787 reads (see bioinformatic analyses section) for the 18 samples.

Shotgun metagenomic analysis was performed on samples belonging to the third year (SP3-1, SP3-2, SP3-3; AU3-1, AU3-2; AU3-3). We repeated the DNA extraction several times to reach the approximate concentration of 500 ng to 1 μg of DNA for each sample. Random shotgun metagenomics was performed in the Unity of Genomics of Scientific and Technological Centers, University of Barcelona (CCiTUB). Number of sequences ranged from 61370 to 140208. Major scaffold distribution lengths were 390–470 bp.

We combined 16S rRNA amplicons, PICRUSt and shotgun metagenomics using the best of each method to obtain the maximal information to try to describe precisely the taxonomical structure and functionality of the samples. The advantages of using 16S rRNA amplicons sequencing had normally better taxonomic resolution than shotgun metagenomics (Tessler et al., 2017), and the availability of bioinformatic tools for prediction of functions (PICRUSt) is particularly attractive to microbial ecologists as it allows them to study the genes (functions) of complex microbial communities with reasonable accuracy at a high taxonomic resolution (Mukherjee et al., 2017). Random shotgun sequencing of environmental DNA provides a direct and potentially less biased view of the functional attributes of microbial communities (Klatt et al., 2013). 16S rRNA gene regions recovered from the shotgun metagenomic data can span the entire length of the genes; the PCR-based amplicon approach only targets the V1–V2 region. Therefore, the two approaches may not necessarily give identical results (Fierer et al., 2012).

### Bioinformatics Analyses

For 16S rRNA amplicons, the raw data of each sample was preprocessed for demultiplex and quality control using a pipeline implemented in GPRO version 1.1 (Futami et al., 2011). Raw reads that contained < 150 nucleotides in size, ambiguities > 1, homopolimer > 8, as well as redundant sequences were removed from each metagenome dataset using screen.seqs and unique.seqs by Mothur1.31.2 (Schloss et al., 2009). Sequences were taxonomically classified using Silva database^1^ (Quast et al., 2013). CD-HIT-EST from the CD-HIT 4.5.4 package (Fu et al., 2012) was used to define clusters of clones within each metagenome with a distance threshold of 0.03 (resulting in a cutoff at the species level). Alpha and beta diversity analyses of all samples were performed at 97% distance level of OTU. For diversity we rarified (normalized) samples to compare all the samples. Weighted UniFrac metrics was used to measure beta-diversity and to generate principal coordinates analysis plots, using the normalized OTU table. For the heatmap we used the OTU table at 0.10% genetic distance level (resulting in a cutoff at the family level) (Yarza et al., 2010), and make_otu_heatmap.py, and the script was modified by Stamp program. Hierarchical cluster analysis used for similarity measure was Pearson’s correlation, and for the clustering algorithms, Ward’s linkage.

Core microbiota was determined using compute_core_microbiome.py in qiime^2^ (Caporaso et al., 2010). Core OTUs were defined as the OTUs that are present in at least 90% of the samples. From the set of OTUs that could be considered the core, we performed an ecological network of interactions. Ecological network was achieved by Molecular Ecological Network Approach (MENA)^3^ (Deng et al., 2012). Ecological network worked with RMT (random matrix). To visualize the network it was used Cytoscape 3.5.1.

Metagenomes were predicted from the 16S rRNA data using PICRUSt (Langille et al., 2013) for samples corresponding to years 1, 2, and 3. This was prepared by the predict_metagenomes.py script against functional database of KEGG Orthology. Functional contributions of various taxa to different KOs were computed with the script metagenome_contributions.py (Mukherjee et al., 2017). For the third-year samples, gene annotation of the shotgun method was analyzed by the United States Department of Energy Joint Genome Institute^4^ (Nordberg et al., 2014).

The DOE-JGI Metagenome Annotation Pipeline (MAP) supports the annotation of metagenomic sequences and it is organized in three stages: sequence data pre-processing, structural annotation, functional annotation and phylogenetic lineage prediction. Some of the processing methodology used by MAP was as follows: Unassembled 454 reads containing more than five occurrences of ‘N’s are removed. Sequences shorter than 150 bp after trimming are also removed. When two or more sequences are at least 95% identical, with their first 3 bp being identical as well, those sequences are considered to be replicates and only the longer copy is retained. For genomic assembler it is used the Velvet algorithm package. A good kmer size is just over half a read length, which prevents sequencing errors from forming bubbles. Ribosomal RNA genes (5S, 16S, 18S, 23S) are predicted using hmmsearch tool from the package HMMER 3.1b2. The pipeline runs against curated models, derived from full-length genes within IMG, while keeping the best scoring models. The identification of protein-coding genes is performed using a consensus of four different *ab initio* gene prediction tools: prokaryotic GeneMark.hmm (v.2.8), MetaGeneAnnotator (v. Aug 2008), Prodigal (v. 2.6.2) and FragGeneScan. Protein-coding genes with translations shorter than 32 amino acids are deleted. Assignment was made at 90% of the KO gene sequence that was covered by the alignment (Huntemann et al., 2016).

The numbers of the analysis projects in the JGI were Ga0197827, Ga0197828, Ga0197830, Ga0197833, Ga0197836, Ga0197838. For the 16S rRNA amplicons, sequence data were deposited on the NCBI database by the Bioproject PRJNA416849.

## Results

### Phylogenetic Stratigraphy in the Camargue Microbial Mats

Camargue microbial mats composition of Bacteria, Archaea and Eukarya were based on data obtained by shotgun metagenomics. Microbial communities were dominated by Bacteria (92.4–94%), while Archaea and Eukarya represented 4–5% and 1–1.6%, respectively. The distribution of Archaea phyla in spring and autumn was similar but there were several differences across the three depths sampled (0–2, 2–4, and 4–6 mm). Thus, Archaeal relative abundances at those depths were 4.2, 4.6, and 5.1%, respectively. The major phyla were Euryarchaeota (80.6%), followed by Crenarchaeota (8%), *Candidatus* Micrarchaeota (3.9%), and Thaumarchaeota (ammonia-oxidizing archaea, 2.8%).

The eukaryotic diversity of the Camargue microbial mats was sparse, in contrast to the vast bacterial diversity. This was probably due to the broad metabolic capabilities of Bacteria, which enable them to occupy a broad range of chemical niches, whereas the metabolic versatility of eukaryotes is more limited, despite their ability to survive under high sulfide, fermentative, and anoxic conditions. Eukarya represented 1% of the total relative abundance of microorganisms from the mat, with the most representative eukaryotes those related to algae (Chlorophyta), plants (Streptophyta), fungi (Ascomycota), and Arthropoda (insects, mainly *Anopheles*). This result contrasts with the findings in the Guerrero Negro microbial mats, where the dominant eukaryotic organisms are bacterivorous nematodes (Feazel et al., 2008).

**FIGURE 1:**
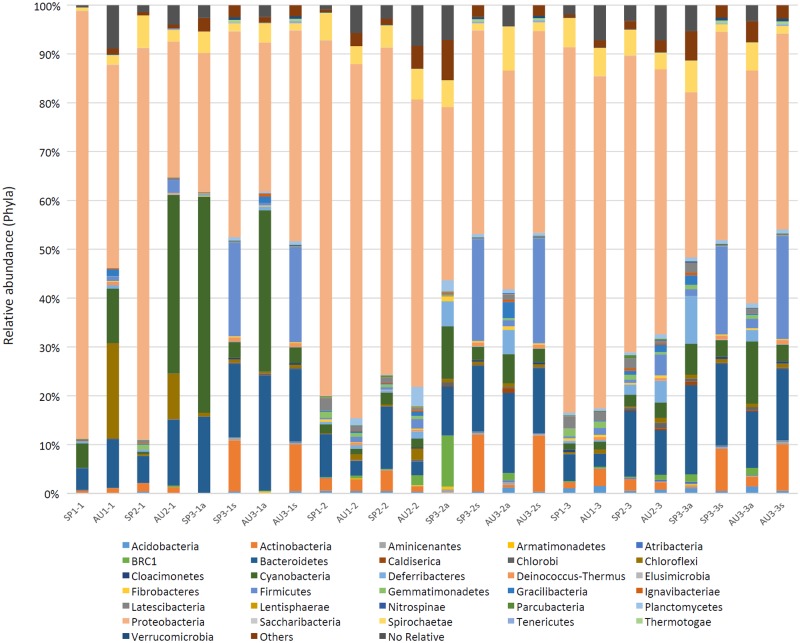
Relative abundances of bacterial phyla from the Camargue microbial mats. Samples SP1-1, SP1-2, SP1-3; AU1-1, AU1-2, AU1-3; SP2-1, SP2-2, SP2-3; AU2-1, AU2-2, AU2-3; SP3-1, SP3-2, SP3-3; AU3-1, AU3-2; AU3-3. SP and AU indicate the season analyzed; the first number, year 1, 2, or 3; and the second number, the layer (1, 0–2 mm; 2, 2–4 mm; 3, 4–6 mm). Samples for the third year were accompanied by a and s, amplicons and shotgun, respectively.

**FIGURE 2:**
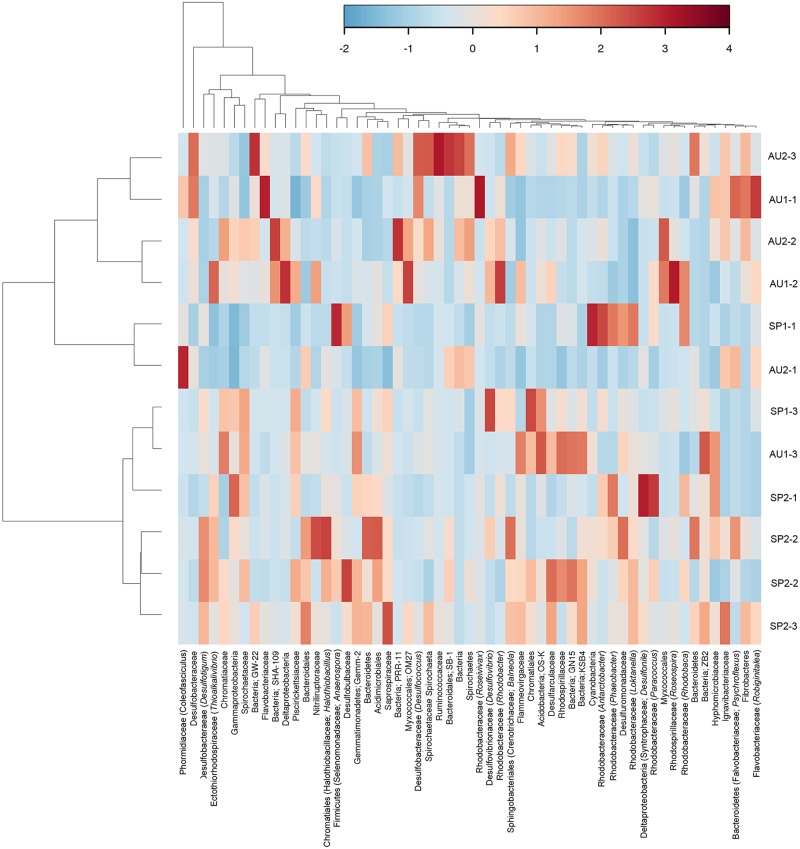
Heatmap and dendogram of the relative abundance of OTU respect to families identified in the Camargue microbial mats. OTUs’ table was done at 0.10% genetic distance level (Yarza et al., 2010). Several representative OTUs could be classified at genus level.

More than 30 phyla of Bacteria were recovered from amplicon sequencing of the 16S rRNA gene and shotgun metagenomics isolated from the Camargue microbial mats. Among the distinguished phyla, there were six that dominated: Proteobacteria (40.2–75.2%), Bacteroidetes (2.6–16.6%), Firmicutes (1.2–21.5%), Actinobacteria (1.0–10.4%), Cyanobacteria (1.2–36.6%), and Spirochaetes (1.4–6.6%) (**Figure 1**). The use of shotgun metagenome analysis in the 3rd-year samples did not yield additional phyla compared to the 16S rRNA amplicons analyzed using Silva database. However, it did detect a relatively high abundance of Actinobacteria, Bacteroidetes, and Firmicutes, and a lower relative abundance of Proteobacteria. The distributions of several phyla and their families depended on the layer analyzed and the season (**Figure 2** and **Supplementary Figures S1A,B**). Cyanobacteria were more abundant in the upper layer (0–2 mm) and in autumn. Alphaproteobacteria were the most abundant Proteobacteria, followed by Gammaproteobacteria and Deltaproteobacteria. Alphaproteobacteria, especially the family *Rhodobacteraceae*, were abundant in the upper layers (0–2 and 2–4 mm) and in the spring. Gammaproteobacteria were represented, in order descendent of relative abundance, by *Chromatiaceae*, *Ectothiorhodospiraceae*, and *Pseudoalteromonadaceae*. The *Chromatiaceace* family was present in all layers with a slightly increased abundance at 2–4 and 4–6 mm, but no difference between spring and autumn. *Ectothiorhodospiraceae* were more abundant in the upper layer (0–2 mm) than in the other, deeper layers. *Pseudoalteromonadaceae* were detected only in the autumn samples. Among the Deltaproteobacteria, *Desulfobacteraceae* was the most abundant family detected and their distribution in the different layers and in the two seasons was similar. *Chloroflexales* (phylum Chloroflexi) were more abundant in the autumn samples and in the upper layer. Several genera, such as *Thioalkalivibrio*, *Desulfotigum*, *Roseovarius*, etc., were detected through the years analyzed, but their distribution depended on the layer (**Figure 2**).

**FIGURE 3:**
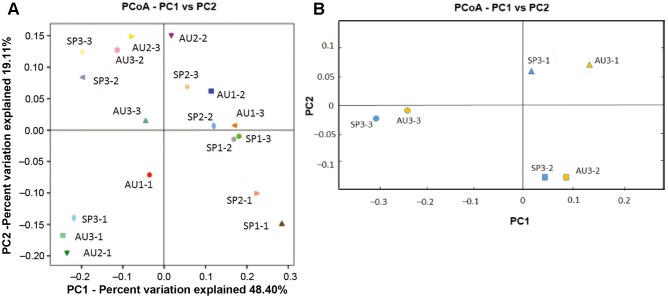
Principal component analysis of the community distribution by year, season and layer from the Camargue microbial mats. **(A)** β-Diversity coupled with principal coordinates analysis was used to compare the bacterial composition in Camargue microbial mats by season and layer. Unifrac weighted was implemented in the QIIME program (Caporaso et al., 2010). Red squares represented the oxic/photic layer; Blue triangles, oxic-anoxic transition layer; Orange, anoxic layer. The phylogenetic P-test in Unifrac, indicated that the microbial communities were not significant different (*P* > 0.05). But pairwise significance tests using the t-Student showed significant differences between oxic/photic layer and the other layers (transition oxic-anoxic layer and anoxic layer). **(B)** Principal coordinates analysis of functional annotation of shotgun metagenomes processed in the JGI database [http://www.jgi.doe.gov/]. To compare the genomes (third year samples), we used the KO genes as a row with significant hits, and with a minimal function gene count for 5. The PCA analysis showed that the PC1 the percent of variation was explained by 18.7% and the PC2, 26.57%. The t-Student had similar results than for the taxonomical results in A.

In the rarefaction curves for samples with respect to depth and season, at 0.03 similarity the samples did not reach an asymptote, suggesting their insufficient sequencing depths (**Supplementary Figure S2**). In our analyses we used the abundance estimator Chao1 and abundance-based coverage estimator (ACE), Shannon and Simpson diversity indexes, and Berger-Parker dominance index (**Table 1**). The highest diversity was found in the third layer (4–6 mm). The surface layer in all samples exhibited the lowest diversity, with a few strongly dominant OTUs, especially photosynthetic bacteria. A principal component analysis indicated that the population community structure of the upper layer (oxic/photic layer) differed from that of the transitional oxic-anoxic layer (2–4 mm) and the anoxic layer (4–6 mm), which were relatively close together (**Figure 3A**). These results were independent of the season and the sampling year. Samples for the oxic/photic layer (0–2 mm) had the most distant community distribution, probably due to the high relative abundances of Cyanobacteria and Chloroflexi. The phylogenetic *P*-test in Unifrac indicated that the microbial communities were not significant different (*P* > 0.05). But pairwise significance tests using the t-Student based on taxa detected showed significant differences between oxic/photic layer and the other layers (transition oxic-anoxic layer and anoxic layer). Significant differences between two samples were based on a 95.0% confidence level. *P* < 0.05 was considered to indicate statistical significance. No significant differences were observed between samples from the same layer. In addition, we could observe a clear difference in distribution respect of the functional annotation KO metagenomes to layers (data from shotgun metagenome for the third year samples) (**Figure 3B**). The t-Student from taxonomical data showed the same results that those just mentioned.

**Table 1:** Diversity and richness indexes at 0.03 distance.

Sample^a^	Raw reads	Clean reads	No. OTUs	Chao 1	Ace	Bergerparker	Shannon	Simpson
SP1-1	7811	4114	205	634.04	856.45	0.127	4.51	0.0330
SP1-2	6656	3453	325	1206	1634.46	0.0588	5.33	0.0126
SP1-3	8285	3664	397	1387.5	1939.02	0.0392	5.71	0.0082
AU1-1	2825	1156	265	505.01	777.53	0.1203	4.38	0.0392
AU1-2	3053	1313	272	566.25	778.05	0.0422	4.89	0.0137
AU1-3	5361	1432	336	839.25	1148.86	0.0616	5.20	0.0121
SP2-1	3560	2042	291	896.01	1197.66	0.0929	5.02	0.0193
SP2-2	5393	2366	316	876.16	1113.66	0.0605	5.26	0.0119
SP2-3	8054	2866	454	1501.38	2265.98	0.0348	5.91	0.0058
AU2-1	3924	1859	282	711.37	1049.50	0.2478	4.13	0.0805
AU2-2	6022	2778	365	923	1190.00	0.0552	5.40	0.0118
AU2-3	4673	2405	463	1757	2591.69	0.0602	5.79	0.0082
SP3-1	3103	1151	423	765	1325.63	0.0417	5.28	0.0045
SP3-2	6728	3252	390	621	1678.86	0.0147	5.68	0.0009
SP3-3	6919	3130	401	878	1693.39	0.0078	5.86	0.0002
AU3-1	4989	2092	412	808	2188.89	0.0147	5.63	0.0011
AU3-2	5269	3053	433	774	1468.0	0.0104	5.81	0.0004
AU3-3	6591	2661	429	962	2188.9	0.014	5.89	0.0005

^a^*SP and AU indicate the season analyzed; the first number, year 1, 2, or 3; and the second number, the layer (1, 0–2 mm; 2, 2–4 mm; 3, 4–6 mm)*.

### Functional Stratigraphy in the Camargue Microbial Mats

To understand the metabolic potential of the Camargue microbial mats and identify their many different functional features, we used PICRUSt (based on 16S rRNA gene amplicon) and random shotgun metagenomics methodologies. The predicted proteins were classified as KEGG orthologs (KOs). The nearest sequence taxon index (NSTI) values is a measure of how closely related the OTUs in each sample are to the reference genomes in the database. In our case, the “nearest sequence taxon index” (NSTI) values per sample ranged for 0.072–0.172. The taxonomical classification could be accurate at family level, in few cases to genera level, but it was difficult to achieve the species level. This result could explain the values observed at NSTI. Respect to the shotgun metagenomics, KEGG pathways via KO (percentage) ranged from 14.85 to 17.17%; and KO genes ranged from 24.97 to 29.30% respect to the number of sequences (total sequences analyzed ranged from 61,370 to 140,208), and assignment was made at 90% of the KO gene sequence that was covered by the alignment (Huntemann et al., 2016).

**FIGURE 4:**
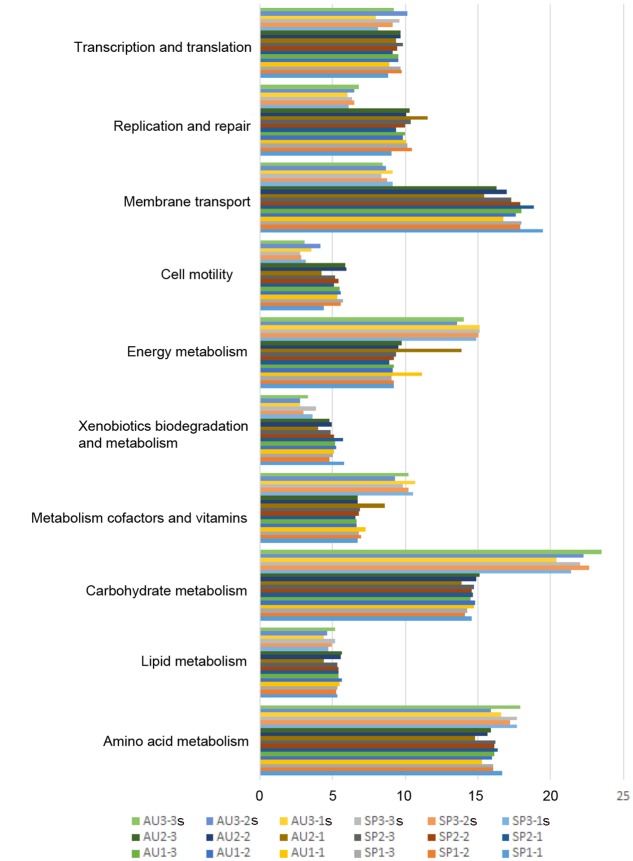
Relative abundances of genes in KEGG classified by biological functional category from the Camargue microbial mats. Functional categories studied were: “metabolism,” “cellular processes,” “environmental information,” and “genetic information processing.” SP and AU indicate the season analyzed; the first number, year 1, 2, or 3; and the second number, the layer (1, 0–2 mm; 2, 2–4 mm; 3, 4–6 mm). For the third year, only data obtained from the shotgun (e.g. SP3-1s, etc.) has been represented.

The biological processes identified were essential for sustaining prokaryotic life in the environment. They include transcription and translation functions (8.7–9.8% relative abundance genes, based on the total number of genes detected in the sample) and replication and repair functions (9–10.2%). Other functional processes were related to cellular processes such as cell motility (4.3–5.9%). Genes related to membrane transport (17.2–19.5%) and to metabolic functions (56.3–58.4%), which included the metabolism of carbohydrates, lipids, amino acid, cofactors and vitamins, xenobiotic biodegradation, and energy metabolism. PICRUSt and shotgun metagenomic analyses revealed similar functional biological processes in all samples analyzed, except of carbohydrate metabolism and energy metabolism, which contained more genes detected by shotgun than by PICRUSt analyses (**Figure 4**).

Gene content analysis provides a basis for inferring the possible metabolic functions of dominant populations present in the community. Cell motility, represented by chemotaxis genes, such as *cherA, cheBR, motA, mcp, pixJ*, etc., peaked at the oxic-anoxic transition zone, but they were also important in the oxic/photic zone. These genes were associated to phototrophic organisms (Cyanobacteria, Alpha-Gammaproteobacteria, and Chloroflexi), but also to heterotrophic members, such as Bacteroidetes and Spirochaetes. Ferredoxins have a negative redox potential act as electron distributors in various metabolic pathways. The genes that codify different ferredoxins were detected in all layers, but they were especially abundant in the oxic-anoxic transition zone. Ferredoxins likely reflected diversification of redox reactions required for respiration (**Supplementary Table S1**). Osmotic regulation is required for microbial survival in hypersaline environments. Accumulation of osmoprotective molecules, in particular glycine betaine, is an adaptive mechanism to pawn the high salinity conditions. We searched for genes implicated on the glycine-betaine biosynthesis, such as *betA, betB, gbsA* (Wong et al., 2015). These genes were distributed through the layers (especially on the oxic/photic layer), and they were associated with different taxa, showing that the microbial mat community could be adapted to salinity conditions (**Supplementary Table S1**).

Genes associated to oxygenic photosynthesis and bacteriochlorphylls were detected in the upper layer (photic zone) in autumn and spring samples (Table S1). Regarding the photosynthetic reaction center in the anoxygenic photho-breaksystem, *pufL* and *pufM* genes were detected and they belonged to Gammaproteobacteria (purple sulfur bacteria, *Chromatiaceae*). The possibility of alternative light energy usage by (bacterio)rhodopsin in different prokaryotic members of the mat cannot be confirmed because we could not detect related genes, even though retinal-based phototrophy could contribute as energy source in layers with low irradiance (Thiel et al., 2017).

In the studied metagenomes we identified the four known autotrophic carbon fixation pathways (the Calvin-Benson cycle, the reverse tricarboxylic acid cycle, the Wood–Ljundahl pathway, and the 3-hydroxypropionate bi-cycle) (Hügler et al., 2002, 2005; Ragsdale and Pierce, 2008; Berg, 2011; Thiel et al., 2017), which suggested the occurrence of a relatively diverse autotrophic community (**Supplementary Table S1**). Debris of predated bacteria by *Bdellovibrionaceae* and viruses may be another carbon source for other heterotrophs. *Bdellovibrionaceae* represented 1–9% of the relative abundance of Deltaproteobacteria. *Bdellovibrionaceae* are predatory bacteria upon a variety of Gram-negative bacteria. Viruses in the mat could be also involved in cell-lysis processes, based on CRISPR systems detected in the metagenomes.

Regarding nitrogen metabolism, we detected genes associated to nitrogen fixation. The oxic/photic zone contained the more diverse and abundant amount of nitrogen fixation genes (**Supplementary Table S1**). For the ammonium oxidation in nitrification, the main enzyme is the ammonia monooxygenase (*amoA*) that is present in both ammonia-oxidizing archaea and ammonium-oxidizing bacteria (Fan et al., 2015). However, *amoA* was not identified in the studied metagenomes. Nitritification provides the oxidant for anaerobic ammonium oxidation (anammox). We examined *hzoA/hzoB* genes because their ubiquity and high expression in anammox bacteria (Planctomycetes) (Hirsch et al., 2011), but no records of those genes were found, although the phylum was detected in the Camargue microbial mats.

Sulfate reduction genes were present in the metagenome dataset and distributed similarly through the different layers (**Supplementary Table S1**). They affiliated to Deltaproteobacteria and Gammaproteobacteria. Sulfur oxidation activity was also found in the Camargue microbial mats, based upon the presence of the enzyme sulfide:quinone oxidoreductase (*sqr* gene).

**FIGURE 5:**
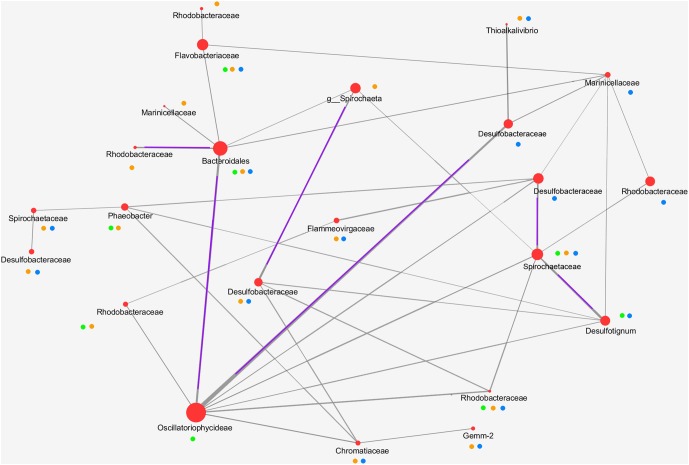
Network showing potential interactions among microbial members of the Camargue microbial mats. Network was constructed by the in base of the “core” community using the Molecular Ecological Network Approach (MENA). Matrix similarity used the Pearson correlation coefficient. Nodes represented the representative OUT, and the size, their relative abundance. The thickness of the lines represented the strength of the correlation between different taxa. These positive interactions were partially marked in the figure with a purple line. Green, orange and blue circles indicated the layer (oxic/photic; oxic-anoxic transition and anoxic layers, respectively) where microorganisms were detected.

Finally, to identify potential biotic interactions within the dominant, prokaryotic communities in the Camargue microbial mats, we constructed a network based on the core OTUs (**Figure 5**). The core OTU were determined by the shared OTUs at 90% in all samples. Several minority populations were not included as the “core community,” and probably they could play important functions. Core community was performed by layer. We observed that there were no significant differences for one layer (e.g., 0–2 mm or 2–4 mm or 4–6 mm) among years and season. In the upper layer (oxic/photic zone), the core microbiota were represented Alphaproteobacteria (*Rhodobacterales*), Gammaproteobacteria (*Marinicellales* and *Chromatiales*—*Ectothiorhodospiraceae*) and Cyanobacteria, (*Coleofasciculus* [formerly, *Microcoleus*], *Oscillatoriales*). In the middle layer (transition oxic-anoxic zone), there were OTUs belonging to Alphaproteobacteria (*Rhodobacterales*), to Gammaproteobacteria (*Marinicellales, Chromatiales, Thiotrichales*), to Deltaproteobacteria (*Desulfobacterales*), to Bacteroidetes (*Flavobacteriales* and *Cytophagales*), to Spirochaetes, and to Cyanobacteria (*Oscillatoriales, Coleofasciculus*). In the bottom layer (anoxic zone), taxons were represented by Alphaproteobacteria (*Rhodobacterales*), OTUs to Gammaproteobacteria (*Marinicellales* and *Chromatiales*), Deltaproteobacteria (*Desulfobacterales*), Bacteroidetes (*Flavobacteriales*. *Bacteroidales* and *Cytophagales*), Spirochaetes, Planctomycetes, and Gemmatimonadetes. The network obtained showed that interactions among taxa could be done through different layers (**Figure 5**). The network was probably incomplete because there were not represented other populations, such as Firmicutes, Actinobacteria, Archaea, or other minor population with less than 0.1% of relative abundance (Planctomycetes, Nitrospinae, Saccharibacteria, etc.), which they could contribute and participate on metabolically interactions within the microbial mat. Cyanobacteria stablished the more diverse interactions with different population’s members of the microbial mat. Positive correlations based the thickness of the lines (in the figure marked by a purple line) and were observed between Cyanobacteria and Deltaproteobacteria; Cyanobacteria and Bacteroidetes; Cyanobacteria and Rhodobacterales; and Spirochaetes and Deltaproteobacteria.

## Discussion

Microbial diversity within an ecosystem has most often been estimated based on the amplification of specific gene targets (e.g., 16S rRNA) and random shotgun sequencing (Klatt et al., 2013; Warden et al., 2016; Cardoso et al., 2017). Our results shed light on the diversity of microbial communities, such as Bacteria (92.4–94% relative abundance), Archaea (4–5%) and Eukarya (1–1.6%), present in the Camargue microbial mats. Although an intrinsic bias of the method cannot be ruled out, as already noted by other authors (Amend et al., 2010; Zhou et al., 2011). Cardoso et al. (2017) found differences in taxonomic assignment based on whether the variable region of 16S rRNA V1–V3 vs. 16S rRNA V3–V4 sequences derived from the DNA template. They detected a higher abundance of Proteobacteria using the V1–V3 than the V3–V4 region, whereas the abundances of Bacteroidetes, Chloroflexi, and, particularly, some rare phyla were lower using the V1–V3 dataset. Nonetheless, the amplicon sequencing and shotgun metagenomics data obtained in this study confirmed the importance and numerical dominance of Proteobacteria in the Camargue microbial mats as well as in mats from elsewhere in the world (Ley et al., 2006; Ruvindy et al., 2016; Warden et al., 2016; Cardoso et al., 2017). Proteobacteria participate in the sulfur cycle, especially the purple sulfur bacteria belonging to the Gammaproteobacteria, purple non-sulfur bacteria belonging to the Alphaproteobacteria, and the sulfate-reducing bacteria belonging to the Deltaproteobacteria (Bolhuis et al., 2014; Ruvindy et al., 2016).

The distribution of Archaea phyla in spring and autumn was similar but there were several differences across the three depths. Euryarchaeota at the surface were dominated by the classes *Halobacteria* and *Methanomicrobia,* and in the deeper layers by *Methanobacteria*, *Halobacteria*, *Methanomicrobia*, and *Methanococci* (in order of their relative abundances). The dominance of Euryarchaeota was also described for other hypersaline microbial mats, except Guerrero Negro, which is dominated by Crenarchaeota (Robertson et al., 2009; Schneider et al., 2013; Fernandez et al., 2016; Wong et al., 2017).

Eukarya represented 1% of the total relative abundance of microorganisms present in the mat. The eukaryotic diversity of the Camargue mat was sparse, in contrast to the vast bacterial diversity. This was probably due to the broad metabolic capabilities of Bacteria, which enable them to occupy a broad range of chemical niches, whereas the metabolic versatility of eukaryotes is more limited. Also, some environmental factors such as salinity, oxygen and sulfide gradients could be limiting factors for the eukaryotic diversity. Halophiles are found in all three domains of life and they are components of brine communities. Within the Bacteria: *Cyanobacteria*, *Proteobacteria*, *Firmicutes*, *Actinobacteria*, *Spirochaetes*, and *Bacteroidetes*. Within the Archaea: *Halobacteria*, and for eukaryotes, Alveolates (ciliates and dinoflagellates), several Fungi (e.g., Wallemia, *Trimmatostroma, Hortaea*), chlorophytes, Euglenozoans, shrimp (e.g., *Artemia)* (Oren, 2008). Salinity (only) probably is not a limiting environmental factor for the development of eukaryotes, but their combination with daily changes in oxygen and sulfide may affect their survival in microbial mats.

Cyanobacteria were detected in the Camargue microbial mats in relative low numbers and they were dominated by the species *Coleofasciculus* (formerly *Microcoleus*) *chthonoplastes* (*Oscillatoriales*). This result may be a consequence of the methodology used, as the efficiency of cell lysis strongly varies among different microorganisms. Filamentous Cyanobacteria are heavily encapsulated by exopolysaccharides (EPS) and therefore they are difficult to lyse. Moreover, even when lysis is successful, nucleic acids may become trapped in the EPS and thus inaccessible for PCR and sequencing (Bolhuis et al., 2014). Ramos et al. (2017) reported that studies using Cyanobacteria-specific primers rendered high cyanobacterial diversity. However, the scarcity of cyanobacteria and their low diversity have been described in several mats (Ley et al., 2006; Fernandez et al., 2016).

In microbial mats, the import and export of microorganisms are low and the community composition is accordingly stable (Cardoso et al., 2017). The environmental conditions, including temperature and salinity, during the sampling period were not sufficiently different to significantly modify the microbial communities. Rather, vertical gradients of light and redox (oxic-anoxic) conditions were the likely determinants of mat community structure. In the presence of oxygen land high light intensity (oxic/photic zone, 0–2 mm), the prokaryotic communities in the surface layers were mainly composed of Cyanobacteria and anoxygenic phototrophs (Alphaproteobacteria, represented mainly by purple non-sulfur bacteria, *Rhodobacterales*, and *Rhodospirillaceae*). However, while *Rhodobacterales* species may prosper in the surface layer of the mat, most *Rhodospirillaceae* prefer anoxic conditions (Schneider et al., 2013). Archaea in the surface layer were represented by *Candidatus* Micrarchaeota, ammonia-oxidizing Thaumarchaeota, and Euryarchaeota (*Halobacteria*). *Halobacteria* uses bacteriorhodopsin to transform light energy into chemical energy by a process unrelated to chlorophyll-based photosynthesis. Chemotaxis and motility genes were assigned to phototrophs, such as Cyanobacteria, purple sulfur bacteria, and purple non-sulfur bacteria, consistent with the ability of these microorganisms to search for optimal environmental conditions, including light. The surface layer in all samples exhibited the lowest diversity, with a few strongly dominant OTUs, especially photosynthetic bacteria as observed by other authors (Armitage et al., 2012; Al-Najjar et al., 2014; Bolhuis et al., 2014). Upper layers can have extreme physicochemical conditions if the mat is desiccated, during the day it may have high light irradiance, temperature, and high salinity due to water evaporation. In our case, mats were flooded all seasons (cover by ca. 10–20 cm of water). Cyanobacteria were the major phylum at the top layer and they may be adapted to those conditions (Bolhuis et al., 2014; Al-Najjar et al., 2014; Pade and Hagemann, 2015).

The transition zone (2–4 mm) contained Alphaproteobacteria (purple non-sulfur bacteria), Gammaproteobacteria (*Chromatiaceae* —such as *Thiohalocapsa* and *Halochromatium*— were more abundant than *Ectothiorhodospiraceae —*such as *Thioalkalivibrio—*), *Candidatus* Chlorothrix, which was the most abundant genus of green non-sulfur bacteria of *Chloroflexales* (Chloroflexi), Deltaproteobacteria (sulfur reducing bacteria), and heterotrophic fermenting bacteria. In the anoxic zone (4–6 mm), Deltaproteobacteria and fermenters, especially Spirochaetes, comprised the major part of the bacterial community.

Microorganisms detected through layers could interact metabolically by the different genes detected that worked in carbon, nitrogen and sulfur nutrients cycles, being a self-sustaining system. Producers, such as photosynthetic microorganisms contributes to the nourishment of heterotrophs members of the community. Cyanobacteria (mainly *Coleofasciculus*) must provide a source of carbon to the heterotrophs, and a source of H_2_ for sulfate-reducing bacteria (Deltaproteobacteria, such as *Desulfonema*, *Desulfotigum*, *Desulfococcus*, *Desulfonile*) (Lee et al., 2014). In the dark, cyanobacteria fermented their carbon reserves excreting low-molecular-weight organic acids and hydrogen (Hoffmann et al., 2015). Hydrogen can be utilized as electron donor by the anoxygenic photosynthetic bacteria (Nielsen et al., 2015). Nutritional interdependence among microbial populations is exemplified by an anaerobic community operating from hydrolytic to fermenting primary anaerobes, then to syntrophic bacteria and to homoacetocetic, methanogenic, or sulfidogenic secondary anaerobes. In diverse anoxic environments, spirochetes occupy an intermediate trophic level between the hydrolytic bacteria and these secondary anaerobes; this is because the main compounds produced by spirochete are acetate, H_2_, and CO_2_, which are normally consumed by sulfate-reducing bacteria and methanogens (Blazejak et al., 2005; Berlanga et al., 2008). In microbial mats, sulfate-reducing bacteria outcompete methanogens because of the high concentration of sulfate in the seawater.

Members of Bacteroidetes (such as *Psycroflexus, Robiginitalea*) were present in all samples. Bacteroidetes are able to grow under a wide range of physicochemical conditions (Farías et al., 2014; Wong et al., 2016) and to degrade polymeric compounds (Fernández-Gómez et al., 2013; Hania et al., 2017). Therefore, Bacteroidetes may play a key role in the degradation and cycling of mat carbon compounds. The family *Rhodothermaceae*, and especially the genus *Salinibacter*, has been detected in abundance in the upper oxic/photic zone in several microbial mats because the respective genera are halophilic and can use light as an additional energy source for growth (Sahl et al., 2008; Schneider et al., 2013). However, in the Camargue microbial mats their relative abundance was low.

Identifying microbes responsible for particular environmental functions is challenging. Microbial mats harbor different microbial symbiont populations with specialized functionalities. In this analysis, we described metabolic potentials and putative interactions among mat community members, leading to an initial overview of the metabolic potential of the entire mat community.

## Author Contributions

MB designed the work. MB and MP performed the experiments and analysis. All authors discussed and interpreted the results. MB and RG wrote the paper. All authors read and approved the final version of the manuscript.

## Conflict of Interest Statement

The authors declare that the research was conducted in the absence of any commercial or financial relationships that could be construed as a potential conflict of interest.

## Footnotes

1 http://www.arb-silva.de

2 http://qiime.org/scripts/compute_core_microbiome.html

3 http://ieg2.ou.edu/MENA

4 http://www.jgi.doe.gov/

## Supplementary Material

FIGURE S1 Relative abundance of families respect on their phyla. **(A)** Relative abundance of Families detected in Bacteroidetes and relative abundance of subphyla from Proteobacteria. **(B)** Relative abundance of several families belonged to Alphaproteobacteria, Deltaproteobacteria and Gammaproteobacteria.

FIGURE S2 Rarefaction curves from 16S rRNA amplicons from 18 samples. Rarefaction was done at 97% identity, and it was normalized by the number of sequences of the smaller dataset.

TABLE S1 Key enzymes for functionality metagenomes.
