# Supplementary figures and images for: Functional Stability and Community Dynamics during Spring and Autumn Seasons Over 3 Years in Camargue Microbial Mats
Source: Front Microbiol. 2017 Dec 22;8:2619. doi: 10.3389/fmicb.2017.02619 (PMC5744480; doi:10.3389/fmicb.2017.02619)

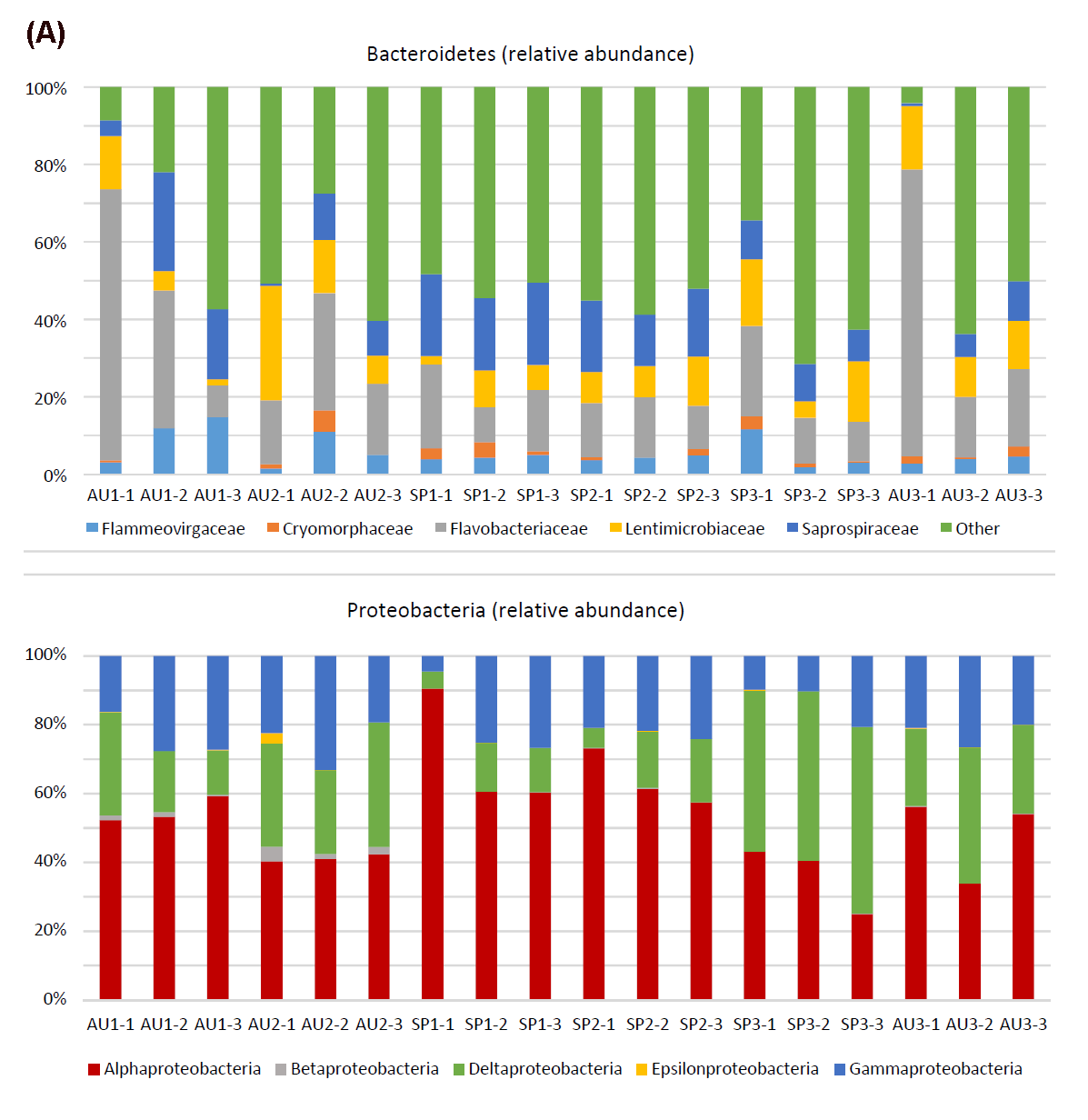

Supplement: FIGURE S1 — Relative abundance of families respect on their phyla. (A) Relative abundance of Families detected in Bacteroidetes and relative abundance of subphyla from Proteobacteria. (B) Relative abundance of several families belonged to Alphaproteobacteria, Deltaproteobacteria and Gammaproteobacteria. [file Image_1.TIF]

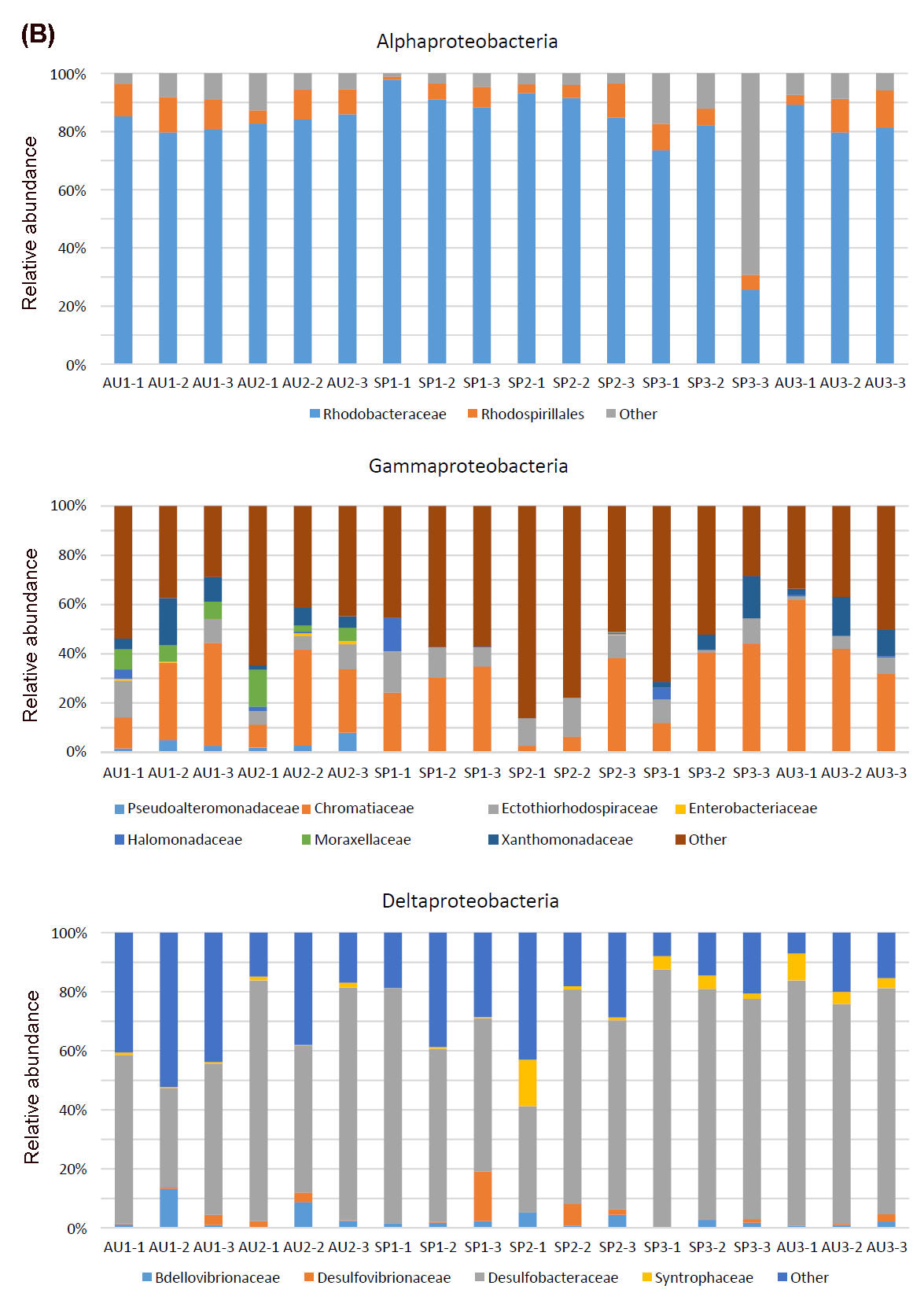

Supplement: FIGURE S2 — Rarefaction curves from 16S rRNA amplicons from 18 samples. Rarefaction was done at 97% identity, and it was normalized by the number of sequences of the smaller dataset. [file Image_2.TIF]

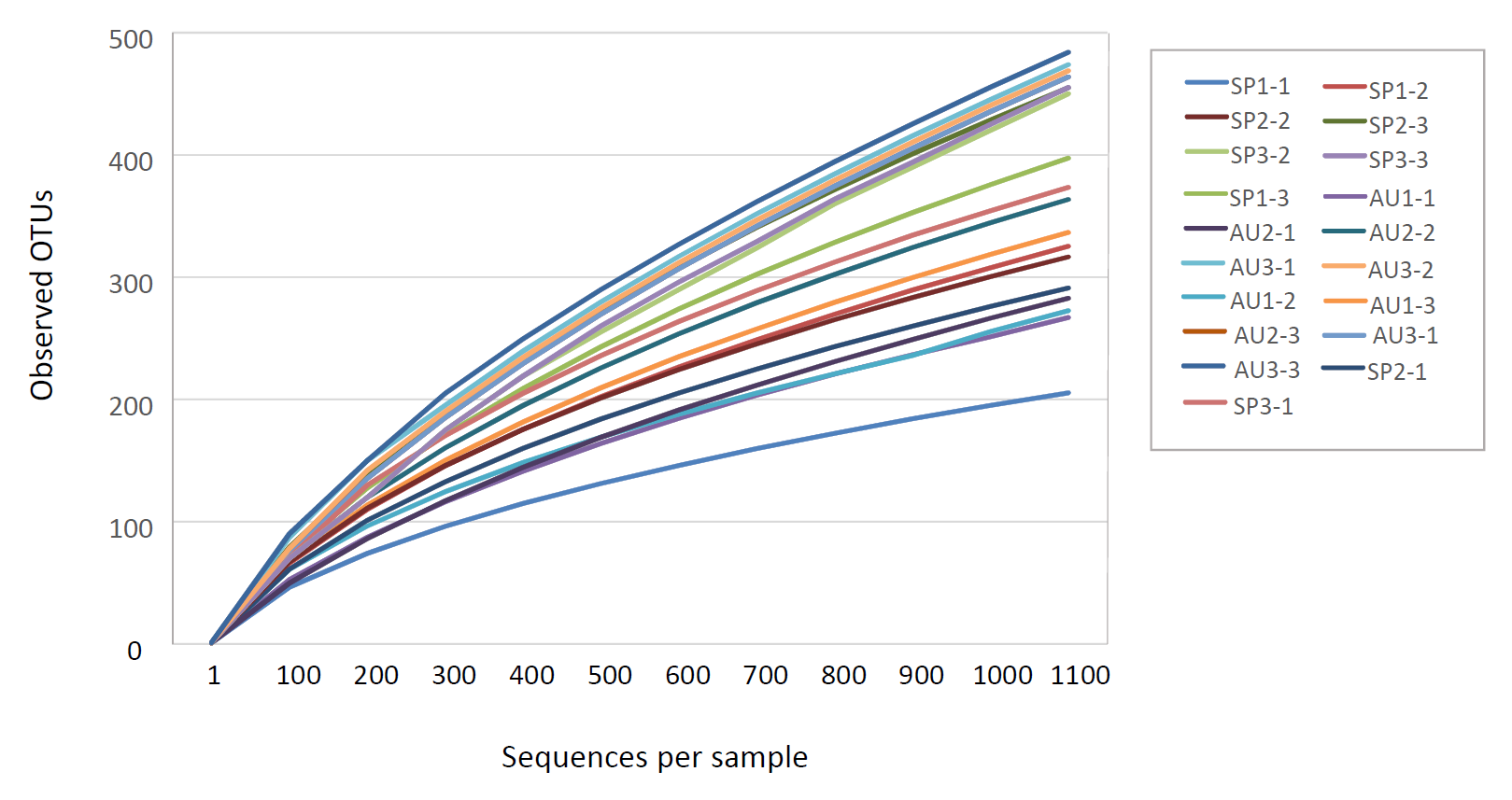

Supplement: Supplementary file 3 [file Image_3.TIF]
