# Supplementary material for: Functional Stability and Community Dynamics during Spring and Autumn Seasons Over 3 Years in Camargue Microbial Mats
Source: Front Microbiol. 2017 Dec 22;8:2619. doi: 10.3389/fmicb.2017.02619 (PMC5744480; doi:10.3389/fmicb.2017.02619)
Supplement: TABLE S1 — Key enzymes for functionality metagenomes. [file Table_1.DOCX]

Table S1 Key enzymes for functionality metagenomes.

|  |  | Gene count | | |  |
| --- | --- | --- | --- | --- | --- |
| KO | gene | 0–2 mm | 2–4 mm | 4–6 mm | Taxonomical groups |
| Chemotaxis | | | | | |
| K00575 | *cheR* | 91 | 110 | 39 | Cyanobacteria, Alpha-Gammaproteobacteria, Chloroflexi, Bacteroidetes and Spirochaetes |
| K02557 | *motB* | 27 | 36 | 18 |  |
| K03407 | *cheA* | 225 | 191 | 74 |  |
| K03413 | *cheY* | 44 | 60 | 14 |  |
| Glycine betaine biosynthesis (osmolarity resistance) | | | | | |
| K00108 | *betA* | 22 | 16 | 12 | Alphaproteobacteria (*Rhodobacteraceae*), Cyanobacteria, Deltaproteobacteria, Chloroflexi, Bacteroidetes, and unclassified |
| K00130 | *betB, gbsA* | 16 | 18 | 8 |  |
| Bacteriochlorophyll |  |  |  |  |  |
| K11336 | *bchF* | 6 | 6 | 5 | Alphaproteobacteria (*Rhodobacteriaceae*) and Chloroflexi |
| K11337 | *bchC* | 15 | 8 | 1 |  |
| K13602 | *bchR* | 10 | 0 | – |  |
| Photosynthesis |  |  |  |  |  |
| K02689 | *psaA* | 21 | 1 | – | Cyanobacteria (*Coleofasciculus chthonoplastes, Leptolyngbya*, *Fischerella* and *Synechococcus*) |
| K02703 | *psbA* | 51 | 5 | – |  |
| Carbon fixation |  |  |  |  |  |
| Calvin benson |  |  |  |  |  |
| K01601 | *rbcL* | 28 | 2 | – | Cyanobacteria (*Coleofasciculus chthonoplastes, Gloeobacter, Gloeomargarita*), Verrucomicrobia (*Verrucomicrobium*), Gammaproteobacteria (*Thiomicrospira*), Actinobacteria; Euryarchaeota (*Ferroglobus*) |
| K01602 | *rbcS* | 3 | – | – |  |
| 3-Hydroxypropionate (3-HP) Bi-Cycle |  |  |  |  |  |
| K14468 | *mcr* | 5 | 4 | 1 | Gammaproteobacteria (e.g., *Pseudohaliea*; *Marinobacter*), Alphaproteobacteria (*Sphingomonadaceae*, *Rhodobacteraceae* ), Bacteroidetes (e.g., *Tangfeifania*; *Saprospira*; *Draconibacterium*; *Psychroflexus*; *Marinilabilia*; *Rikenella*) Cyanobacteria (*Coleofasciculus* *chthonoplastes*); Chloroflexi (e.g., *Oscillochloris; Ardenticatena, Roseiflexus*); Epsilonproteobacteria (e.g., *Sulfurospirillum*); Firmicutes (e.g., *Staphylococcus*); Verrucomicrobia (e.g., *Candidatus* Udaeobacter) |
| K08691 | *mcl* | 1 | 3 | 3 |  |
| K01961 | *accC* | 54 | 56 | 34 |  |
| reverse TCA cycle |  |  |  |  |  |
| K15234 | *ccl* | 1 | 3 | 3 | Firmicutes (Clostridia); Deltaproteobacteria (e.g., *Candidatus* Desulfofervidus auxilii); Aminicenantes; unclassified |
| K15230 | *aclA* | 3 | 2 | 1 |  |
| K15231 | *aclB* | 1 | – | 3 |  |
| Wood-Ljungdahl Pathway (and acetogens*) |  |  |  |  |  |
| K14138 | *acsB* | 7 | 35 | 13 | Deltaproteobacteria (*Desulfohalobiaceae, Desulfobacteraceae*); Chloroflexi; Firmicutes (Clostridia-*Ammonifex*) |
| K00198 * | *cooS, acsA* | 22 | 38 | 43 | Spirochaetes; Deltaproteobacteria (*Desulfosarcina, Desulfococcus, Desulfatiglans, Desulfonatronospira, Desulfobacterium, Desulfomonile, Desulfobaca*); Clostridia (*Desulfovirgula, Acetoanaerobium, Thermacetogenium*) |
| Nitrogen fixation |  |  |  |  |  |
| K02588 | *nifH* | 15 | 6 | 7 | Cyanobacteria (*Coleofasciculus* *chthonoplastes*), Chloroflexi (*Roseifleus,* *Oscillochloris*), Deltaproteobacteria (*Desulfobulbaceae*, *Desulfobulbus*), Gammaproteobacteria (*Thiotrichales*, *Chromatiales*), Alphaproteobacteria (*Rhodobacterales*), Bacteroidetes (*Cytophagaceae*), Spirochaetes (*nif N* gene). |
| K02585 | *nifB* | 29 | 14 | 9 |  |
| K02592 | *nifN* | 13 | 13 | 1 |  |
| Nitrate reduction |  |  |  |  |  |
| K00367 | *narB* | 8 | 8 | – | Cyanobacteria (*Coleofasciculus*, *Geitlerinema*, Nostocales), Alphaproteobacteria (*Rhodobacteraceae*-*Sulfitobacter*) |
| K00371 | *narH* | 4 | 3 | 6 |  |
| nitrite reductasa |  |  |  |  |  |
| K00362 | *nirB* | 12 | 17 | – | Deltaproteobacteria (*Desulfospira, Pelobacter, Desulfohalobium, Desulfobacula*), Clostridia (*Clostridium, Halanaerobium, Caldanaerobius*) |
| K03385 | *nrfA* | 16 | 9 | 5 |  |
| Sulfate Reduction |  |  |  |  |  |
| K11180 | *dsrA* | 14 | 6 | 10 | Deltaproteobacteria (*Desulfonema, Desulfococcus, Olavius, Desulfosarcina*,  Syntrophobacterales), Gammaproteobacteria (*Thioploca*, *Thiocapsa*) |
| K00394 | *aprA* | 6 | 19 | 24 |  |
| Sulfur Oxidation |  |  |  |  |  |
| K17218 | *sqr* | 40 | 21 | 20 | Bacteroidetes (*Marinilabiliales, Flavobacteriaceae*-*Mesoflavibacter*, *Lentimicrobiaceae-Lentimicrobium*), Actinobacteria (*Actinomadura*), Firmicutes (Clostridia-*Desulfosporosinus*), Deltaproteobacteria (*Desulfurivibrio*), Gammaproteobacteria, Cyanobacteria, Alphaproteobacteria (*Rhizobium*, Rhodobacteraceae), Chlorobi (*Chlorobium*) |
| Ferredoxins^a^ |  |  |  |  |  |
| K03738; K00174, etc. | *aor; korA, oorA, oforA; etc* | 718 | 876 | 472 | Diverse taxa, such as, Bacillaceae (Caldalkalibacillus), Clostridiales (Desulfotomaculum, Alkaliphilus, Desulfitibacter ), Detaproteobacteria (Desulfotignum, Desulfoglaeba, Desulfobacterium), Euryarchaeota (Candidatus Methanoperedenaceae), Actinobacteria (Mycobacterium), Synergistetes (Acetomicrobium) |
| – record no found; ^a^Ferredoxins: K00170, K00171, K00174, K00175, K00176, K00177, K00179, K00180, K00187, K00284, K00366, K00367, K00392, K00528, K03737, K05337, K05371, K05524, K05710, K11389 | | | | | |
